# New Surface Modification of Hydrophilic Polyvinyl Alcohol via Predrying and Electrospinning of Hydrophobic Polycaprolactone Nanofibers

**DOI:** 10.3390/foods13091385

**Published:** 2024-04-30

**Authors:** Kihyeon Ahn, Kitae Park, Kambiz Sadeghi, Jongchul Seo

**Affiliations:** 1 Department of Packaging, Yonsei University, 1 Yonseidae-gil, Wonju-si 26493, Gangwon-do, Republic of Korea; tnekfrlgus@gmail.com (K.A.); rbdnjs0504@yonsei.ac.kr (K.P.); 2 School for Engineering of Matter, Transport and Energy, Arizona State University, 501 E Tyler Mall, Tempe, AZ 85287, USA; kambiz_sadeghi@ymail.com

**Keywords:** predrying, polyvinyl alcohol (PVA), polycaprolactone (PCL), electrospinning, surface modification

## Abstract

Despite the excellent oxygen barrier and biodegradability of polyvinyl alcohol (PVA), its poor physical properties owing to its inherent hydrophilicity limit its application. In this paper, we report a novel surface modification technique for PVA films, involving the control of the predrying conditions (i.e., amount of residual solvent) of the coated PVA film and adjusting the electrospinning process of hydrophobic polycaprolactone (PCL) nanofibers onto the PVA films. The residual solvent of the coated PVA film was varied by changing the predrying time. A shorter predrying time increased the residual solvent content significantly (*p* < 0.05) and the flexibility of the coated PVA film. Moreover, scanning electron microscopy depicted the improved physical binding of hydrophobic PCL nanofibers to the hydrophilic PVA surface with increased penetration depth to the PVA film with shorter drying times. The PVA/PCL composite films with different predrying times and electrospun PCL nanofibers exhibited an apparent increase in the contact angle from 8.3° to 95.1°. The tensile strength of the pure PVA film increased significantly (*p* < 0.05) from 7.5 MPa to 77.4 MPa and its oxygen permeability decreased from 5.5 to 1.9 cc/m^2^·day. Therefore, our newly developed technique is cost-effective for modifying the surface and physical properties of hydrophilic polymers, broadening their industrial applications.

## 1. Introduction

Carbon neutrality is a global goal for more than 130 countries and regions [1,2]. Consequently, clean and eco-friendly packaging materials using biobased and biomass materials are being developed [3]. However, conventional packaging materials available in the market, such as polyethylene, polypropylene, and polyethylene terephthalate, are nonbiodegradable and mostly derived from petroleum [4], thereby posing adverse impacts on nature, especially in marine environments [5]. Additionally, biodegradable packaging material cannot maintain their quality without improving their performance [6,7]. Therefore, the development of affordable, nontoxic, and biodegradable packaging polymers for food packaging is highly recommended [8].

Polyvinyl alcohol (PVA) is a biodegradable polymer widely used in various industries, including textiles, paper, packaging, and medicine. The wide applicability of PVA is attributed to its biodegradability by biological organisms, optical properties, high water solubility, biocompatibility, nontoxicity, chemical and thermal stability, and low processing cost [9,10,11,12,13]. However, the applications of PVA are limited owing to its low water resistance, high water vapor permeability, and inferior thermal properties [14,15]. To address these challenges, numerous approaches, including polymer blending, nanotechnology, and physical and chemical crosslinking [16,17,18,19,20], have been investigated.

Electrospinning is a versatile technique for fabricating fibrous nanomaterials with varying chemical and physical properties [21,22,23]. Nanofibers have a high surface-area-to-volume ratio because of their nanoscale dimensions, which can improve the performance of polymer nanocomposites [24]. In particular, the thermal, mechanical, and electrical properties of the resulting nanocomposites can be enhanced by adding electrospun nanofibers to suitable polymers [25,26,27]. Consequently, nanofibers have great potential for enhancing the mechanical and barrier qualities of food packaging and creating cutting-edge structures for functional and intelligent applications. Various studies have demonstrated the use of electrospinning to directly spread nanofibers onto various substrates, particularly polymer films [28,29,30].

We used a technique involving the electrospinning of hydrophobic polycaprolactone (PCL) nanofibers onto a PVA film surface to improve the physical properties of PVA. PCL is a biodegradable material that is widely used to create nanofiber scaffolds by electrospinning [31,32]. Compared to other biodegradable scaffold materials, such as polylactide acid and polyglycolic acid [33,34,35], PCL exhibits favorable mechanical properties and ease of processing. This technique focused on tailoring the physical properties of the PVA film by incorporating PCL nanofibers. However, the poor interactions between the spread fibers and substrates affected the adhesion and delamination, thereby degrading the physical properties, such as tensile strength and thermal properties [36,37]. Moreover, poor interfacial interactions between the hydrophilic PVA substrate and hydrophobic PCL nanofibers were expected [38]. In this context, numerous studies have been conducted to improve the interfacial interactions between hydrophilic and hydrophobic polymers [39]. In particular, the introduction of a straightforward and environmentally friendly method to improve the properties of PVA to broaden its industrial application has been a key aspect.

Here, we propose a novel surface modification technique to strengthen the physical binding and interaction between PCL nanofibers and PVA film as the substrate. PVA and PCL nanofiber (PVA/PCL) composite films were prepared by the direct electrospinning of PCL onto the surface of the PVA film with different solvent contents, which was controlled by varying the predrying time. The chemical and physical properties of PVA/PCL composite films were investigated to determine their feasibility as surface modifiers for hydrophilic PVA films.

## 2. Materials and Methods

### 2.1. Materials

PVA (degree of hydrolysis: 98.0–99.5%; M_n_ = 74,800 g/mol) was supplied by OCI Co., Ltd. (Incheon, Republic of Korea) to produce PVA films. Deionized water was used as the solvent. PCL (M_n_ = 80,000 g/mol) obtained from Merck Sigma Aldrich Co. (St. Louis, MO, USA) was used as the basic polymer for electrospinning. Chloroform and ethanol were obtained from Daejung Chemicals & Metals Co., Ltd. (Gyeonggi-do, Republic of Korea). 

### 2.2. Sample Preparation

**Scheme 1:**
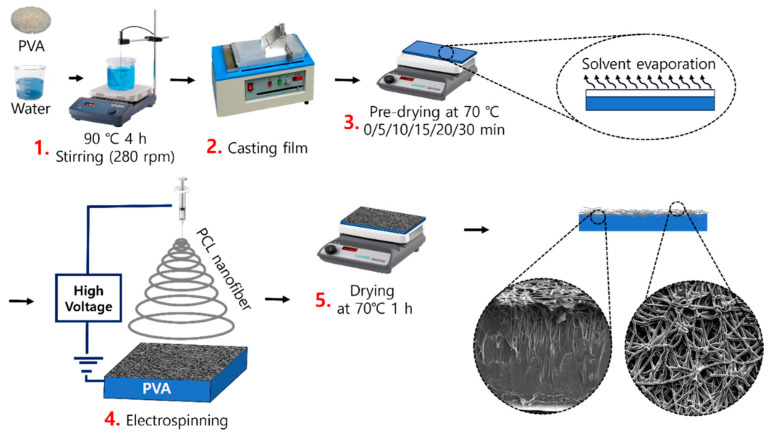
Preparation of the PVA/PCL composite films.

The PVA films were prepared using a solution-casting method. PVA (10 g) was dissolved in water (90 mL) and stirred at 90 °C for 4 h to obtain a homogeneous 10 wt% PVA solution. The PVA solution was used to fabricate films by casting them on a glass substrate. The thickness of the PVA films was approximately 0.11 mm, estimated based on the volume of the PVA solution cast and the known area of the glass substrate. Subsequently, the PVA-coated plates were predried on a hot plate at 70 °C for 0, 5, 10, 15, 20, and 30 min. The PCL nanofibers were produced by electrospinning (ESR200R2, NanoNC Co., Republic of Korea). PCL (7 g) was dissolved at 10% (*w*/*v*) in a chloroform (52.5 mL)/ethanol (17.5 mL) mixture 3:1 (*v*/*v*). The solution was charged at 18 kV and ejected through a 21 G needle at a constant rate of 2.8 mL/h. PCL nanofibers were collected at 15 cm from the surface of the predried film for 30 s and dried on a hot plate at 70 °C for 1 h. The samples obtained with predrying times of 0, 5, 10, 15, 20, and 30 min were named PD-0, PD-5, PD-10, PD-15, PD-20, and PD-30, respectively. Prior to the characterization, the samples were conditioned in a controlled environment at 23 °C and 50% relative humidity for 48 h, following ASTM standard D618 [40] for plastic film conditioning. A schematic of the procedure for preparing the PVA/PCL composite films is shown in Scheme 1.

### 2.3. Characterization

#### 2.3.1. FTIR Analysis

The chemical structures of the samples were determined using an FTIR spectrometer (65 FT-IR, PerkinElmer Co., Waltham, MA, USA) in ATR mode. The analysis was performed in transmission mode for all specimens, encompassing 64 scans within the wavenumber range of 4000–400 cm^−1^. Air was used as the reference for the analysis.

#### 2.3.2. Morphology

The morphologies and microstructures of pure PVA and PVA/PCL composite films were examined by field emission scanning electron microscopy (SEM, JEOL-7800 F, JEOL Co., Ltd., Tokyo, Japan) at an accelerating voltage of 10 kV. Before the analysis, the samples were coated with a thin gold layer using a sputter coating device (Q150R-ES, Quorum Technologies Ltd., Laughton, England).

#### 2.3.3. Water Contact Angle (WCA)

The WCAs of the pure PVA and PVA/PCL composite films were measured using a contact angle goniometer (Phoenix 300, SEO Co., Ltd., Suwon, Republic of Korea). A water droplet was carefully placed on each film. The average WCA was determined by measuring at five different locations on the sample. For each sample treatment, three replications were carried out. The droplets were observed using the θ/2 method with the droplet size approximately at the 20 scale through the eyepiece. The WCA values were measured immediately after the water droplet deposition and after 3 min to consider the capillary effect of the PCL nanofibers, which can enhance the electrolyte absorption and storage capacity [41].

#### 2.3.4. Mechanical Properties

The mechanical properties of the pure PVA and PVA/PCL composite films were evaluated using a QM100T-C universal testing machine (QM 100 T, Omesys Co., Uiwang, Republic of Korea) with a load cell of 20 kgf at a crosshead speed of 20 mm/s according to ASTM D638 standard [42]. For each film, an average of six measurements were conducted, and the resulting mean values were reported.

#### 2.3.5. Thermogravimetric Analysis (TGA)

The TGA of the pure PVA and PVA/PCL composite films were performed using TGA 4000 (TGA-4000, PerkinElmer Co. Ltd., Waltham, MA, USA) in the temperature range of 30–800 °C at a heating rate of 5 °C/min under a nitrogen atmosphere.

#### 2.3.6. Oxygen Transmission Rate (OTR)

The OTR of the pure PVA and PVA/PCL composite films were measured using a gas transmission rate tester (OX-TRAN 702, MOCON Inc., Minneapolis, MN, USA). The OTR of the specimens was determined at the temperature of 23 °C and relative humidity of 0%, according to ASTM D3985 standard [43]. The OTR was carried out with three replications for each sample treatment. 

## 3. Discussion

### 3.1. Effect of Predrying Time on the Residual Solvent and Penetration Depth of PCL Nanofibers onto the PVA Film

**Scheme 2:**
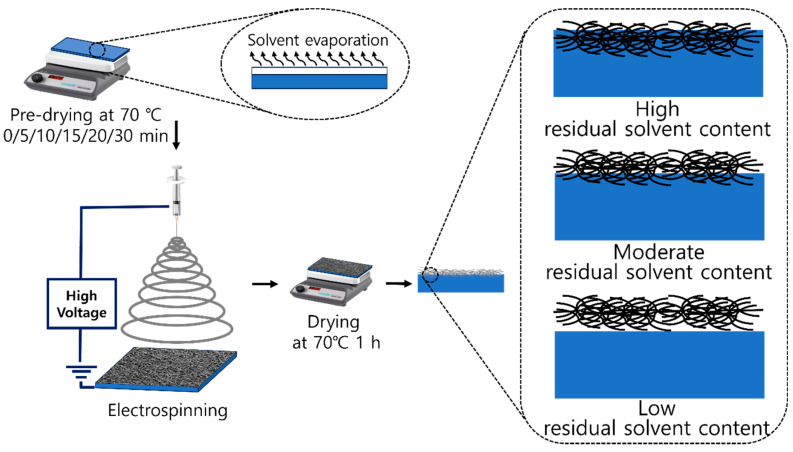
Effect of predrying time on the penetration of PCL nanofibers on PVA films.

Electrospinning is used to directly spread fibers onto various substrates, particularly polymer films. However, the poor interaction between the spread fibers and substrates poses issues with regard to adhesion and delamination, deteriorating the physical properties, such as tensile strength and thermal properties [36,37]. Depending on the polymer chain mobility in the films and post-thermal treatment above the glass transition temperature of the polymer substrate, the spread fibers can be penetrated within the polymer films or settled on the film surface [37,44,45]. Polymer chain mobility is highly dependent on the amount of residual solvent in the polymer films [46], as shown in Scheme 2. High residual solvent content and polymer chain mobility are expected to be favorable for penetrating the spread fibers into the film surface.

**Figure 1:**
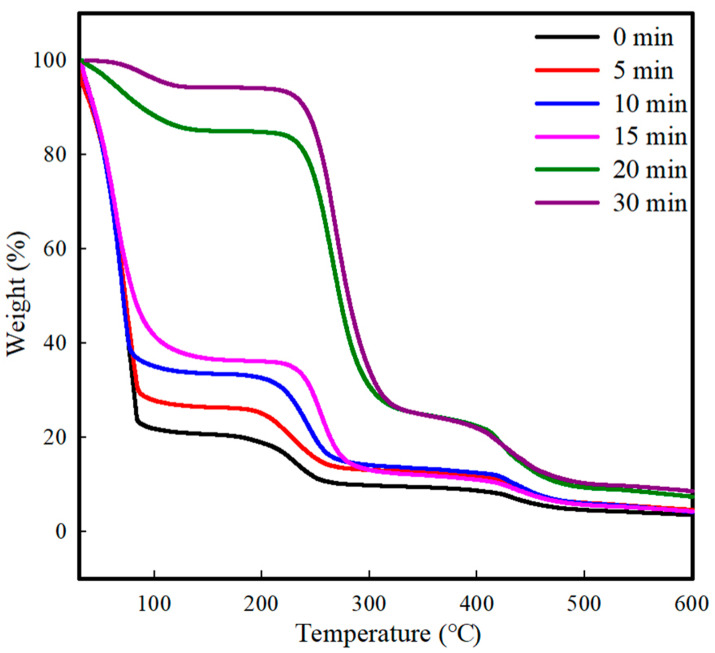
TGA curve of the coated PVA films with different predrying times.

The effect of the residual solvent in the polymer films on the penetration of the electrospun nanofibers was investigated by predrying the coated PVA films at 70 °C for 0–30 min. The amount of residual solvent in the predried PVA films was measured by thermogravimetric analysis (TGA). As shown in Figure 1, the PVA film exhibited three weight loss steps [47]. The first step at temperatures below 160 °C is related to the evaporation of free water and water interacting with the PVA chains. The second step at 200–300 °C is mostly ascribed to the thermal decomposition of the side chains of PVA, and the third step at 400–500 °C indicates the degradation of the main chains of PVA [48]. Although the weight loss pattern did not change with increasing predrying time, the weight loss exhibited a strong dependence on the predrying time. In particular, significant weight loss (*p* < 0.05) was observed with predrying times of 15 min (63.5%) and 20 min (15.0%), as summarized in Table 1. The drying process involves the transport of water as a solvent and the diffusion of water molecules through the interstitial spaces in the PVA film. In our study, a hot plate was used to dry the coated film, in which water molecules diffused from the lower areas of the matrix toward the film surface. Therefore, the water molecules accumulated at the film/air interface increased the mobility on the surface, accelerating the moisture evaporation from the film surface. The different residual solvents in the predried PVA films affected the polymer chain mobility and, consequently, the penetration pattern and penetration depth of the PCL nanofibers electrospun onto the PVA films.

**Figure 2:**
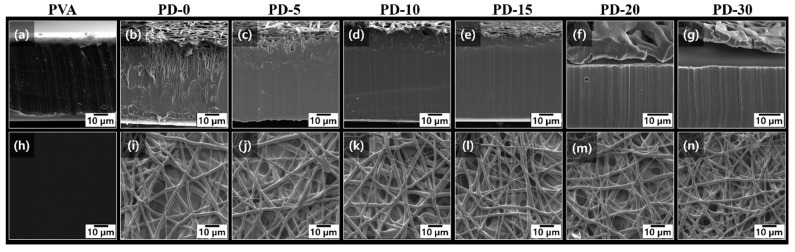
SEM (**a**–**g**) cross-section images and (**h**–**n**) top images of pure PVA and PVA/PCL composite films.

Figure 2 shows the change in the morphology of the surface and cross-section of the PVA films after electrospinning the PCL nanofibers and drying the samples, as investigated by SEM. The pure PVA film cross-section exhibited a planar surface and laminar structure, indicating its typical semicrystalline structure [49]. As shown in Figure 2b–g, the PVA/PCL composite films covered with electrospun PCL nanofibers exhibited a strong dependence on the predrying time and residual solvent content. As expected, PD-0 exhibited the highest penetration depth of the PCL nanofibers. Furthermore, PD-0 showed microvoids inside the PVA matrix, which can be related to the weak interaction between PCL and PVA owing to their different shrinkage rates and hydrophilic/hydrophobic properties [50]. The penetration depth decreased with increasing predrying time. In particular, PD-20 and PD-30, which had low residual solvent contents, did not exhibit any apparent penetration of the PCL nanofibers. As shown in Figure 2f,g, the PCL nanofibers in PD-20 were on the PVA surface or slightly penetrating PVA, whereas the PCL nanofibers in PD-30 were completely delaminated on the PVA surface. These results are related to the residual solvent contents of the predried PVA film. Therefore, a high solvent content in the PVA film increased the polymer chain mobility for PCL nanofibers to penetrate the PVA film. As the penetration depth of the PCL nanofibers can be controlled by optimizing the predrying time, the electrospinning of hydrophobic PCL nanofibers can enhance and modify the surface properties of PVA. Meanwhile, no significant change in topology was observed with different predrying times, as shown in Figure 2i–n. This indicates that sufficient electrospinning time was induced to fully cover the surface of the predried PVA film.

### 3.2. ATR-FTIR 

**Figure 3:**
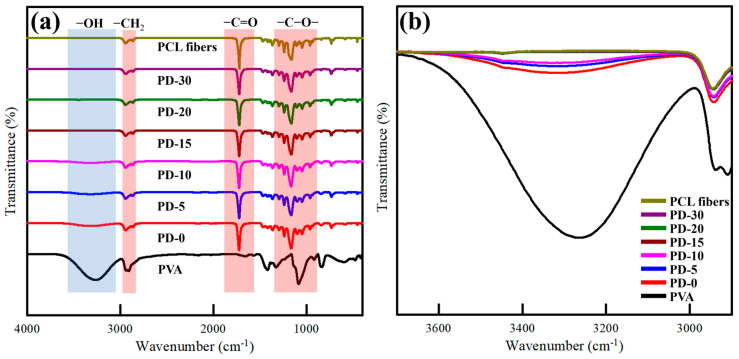
ATR-FTIR spectra of pure PVA, PCL nanofiber, and PVA/PCL composite films (**a**) 4000 to 400 cm^–1^ and (**b**) 3700 to 2900 cm^−1^.

The interaction between the PVA matrix and PCL nanofibers was studied by FTIR spectroscopy (Figure 3a). The distinct transmittance peaks in the pure PVA film were noted at 1720, 2850–2950, and 3000–3600 cm^1^, relating to the C=O, CH_2_, and OH stretching vibrations, respectively [16,18]. PCL nanofiber exhibited several characteristic peaks at 2850–2950 cm^−1^ (–CH_2_ stretching), 1720 cm^−1^ (carbonyl stretching), 1290 cm^−1^ (C–C and C–O stretching), and 1240 cm^−1^ (C–O–C stretching) [51]. All the PVA/PCL composite films showed the same characteristic peaks corresponding to PVA and PCL. Additionally, no obvious peak shift was observed, indicating the absence of strong interactions between PVA and PCL [52]. However, the peak intensity at approximately 3260 cm^−1^ decreased with increasing predrying time (Figure 3b). This suggests that the PVA/PCL composite films without predrying and with predrying for a short period facilitated the deeper penetrating of PCL nanofibers into the PVA film, increasing the hydrophobicity of the PVA film [53].

### 3.3. WCA 

**Figure 4:**
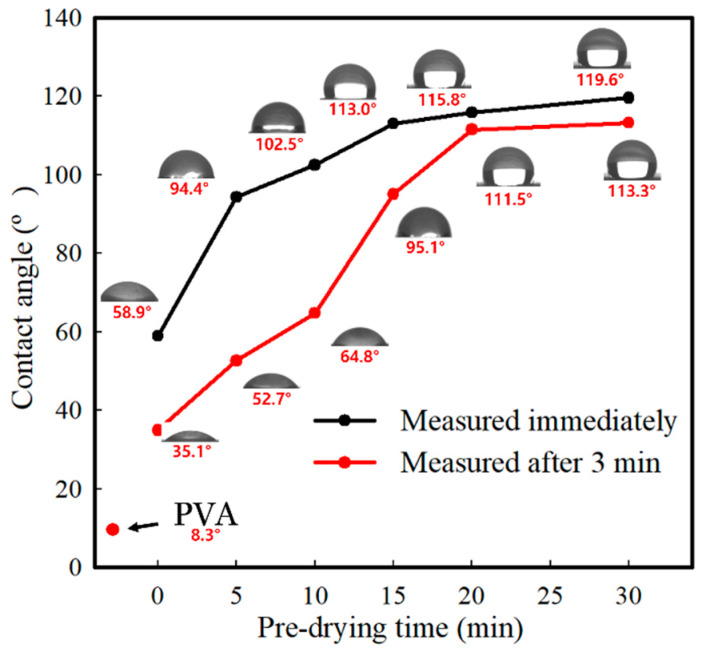
WCA of the pure PVA and PVA/PCL composite films with different predrying times, as measured immediately and at 3 min after the water drop.

The effect of PCL nanofibers and predrying time on the hydrophilic surface of the PVA film was investigated by measuring the WCA, as shown in Figure 4. Pure PVA film showed an inherently low WCA of 8.3°. With the spread of hydrophobic PCL nanofibers, the WCA of the pure PVA film was highly dependent on the predrying time, increasing from 58.9° to 119.6°. Further, the effect of predrying time on the WCA decreased with the predrying time of 15–30 min. However, WCA decreased at 3 min after the water drop, and the rate of decrease was larger for the samples obtained with shorter predrying times. This can be related to the capillary effect of the spread of PCL nanofibers and exposure to hydrophilic PVA. PD-0–PD-10, which have deep penetration of PCL nanofibers into the PVA film, are expected to be more affected by the exposure to hydrophilic PVA, whereas PD-20 and PD-30 are expected to be affected by the capillary effect of the PCL nanofibers laminated on the PVA film. This change was observed only on the top surface (PCL surface-modified layer) and not on the bottom surface (PVA-derived layer).

### 3.4. Mechanical Properties

**Figure 5:**
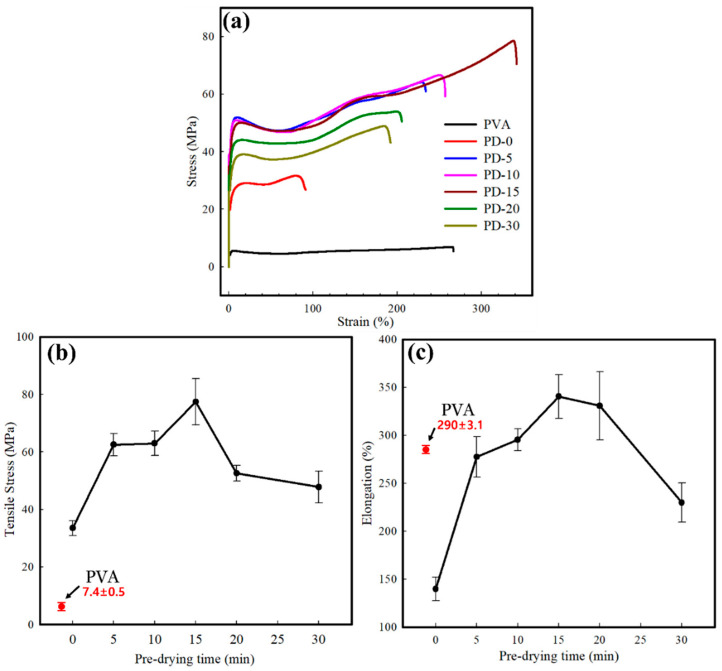
Mechanical properties of the pure PVA and PVA/PCL composite films: (**a**) stress–strain curves, (**b**) tensile strength, and (**c**) elongation at break.

The mechanical properties of the pure PVA and PVA/PCL composite films are shown in Figure 5 and summarized in Table 2. Pure PVA exhibited ductile properties with an elongation at a break of more than 80% [54]. The PVA film exhibited a tensile stress of 7.4 MPa and elongation at a break of 290.0%. The mechanical properties of all the PVA/PCL composite films, including the ductility and tensile strength, were higher than that of the PVA film and highly dependent on the predrying time. Both the tensile strength and elongation at break increased significantly (*p* < 0.05) until a predrying time of 15 min and subsequently decreased with increasing predrying time. Furthermore, PD-0, which had the highest residual solvent content, exhibited a significant decrease in elongation compared with that of the pure PVA film. This could be related to the micropores in the composite film matrix, originating from the poor chemical interactions between the PVA matrix and PCL nanofibers. Several studies have noted an inverse relationship between porosity and elongation [55]. In our study, elongation decreased with increasing porosity in the polymer matrix [55,56]. The elongation of the PVA/PCL composite films with a predrying time of up to 15 min increased and decreased with a further increase in the predrying time. This can be ascribed to the presence of PCL nanofibers, which have a high aspect ratio and good mechanical strength [57]. Moreover, more microvoids were developed in the matrix when the fibers were penetrated deeper, decreasing both the tensile strength and elongation at break [58,59]. Remarkably, PD-15 simultaneously exhibited the highest tensile strength and elongation, contrary to the typical pattern of the inverse relationship between tensile strength and elongation [60]. However, the PCL nanofibers in the PVA matrix, as enhancers of the mechanical properties, can easily slip when the composite film is stretched because of the poor interaction between PVA and PCL nanofibers [61].

### 3.5. Thermal Properties

**Figure 6:**
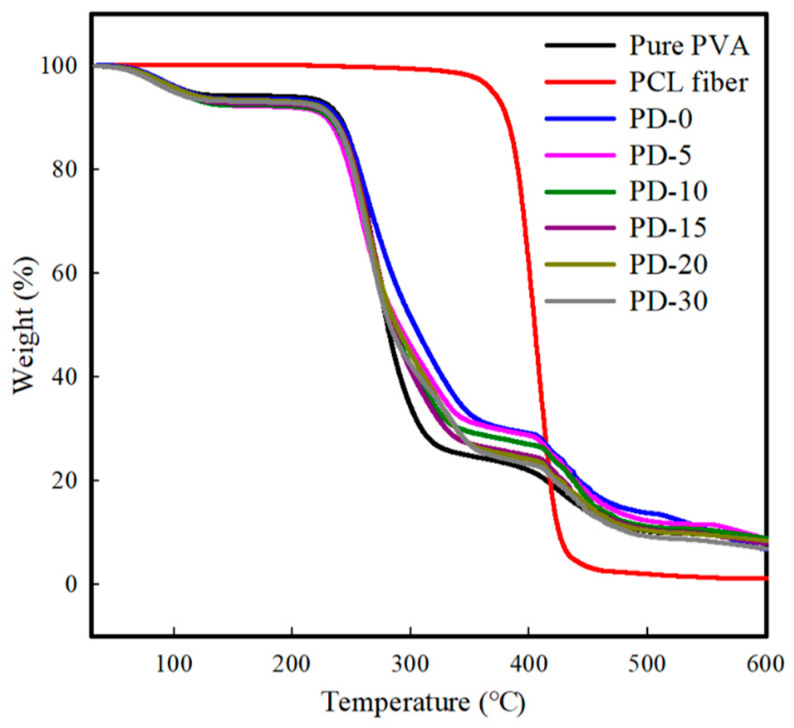
TGA graph of the PCL nanofibers, pure PVA film, and PVA/PCL composite films.

Figure 6 shows the TGA thermograms of the pure PVA and PVA/PCL composite films. The pure PVA film exhibited a general pattern of three weight loss steps [62]. The weight loss steps are related to the loss of the absorbed water molecules at 50–160 °C, loss of water bound to the polymer matrix at 200–340 °C, and decomposition and carbonization of the main polymer chains at 360–500 °C. The PCL nanofibers exhibited one prominent weight loss pattern at 380–460 °C owing to the thermal decomposition of the PCL main chains, indicating higher thermal stability than pure PVA. The PVA/PCL composite films with different penetration patterns and PCL nanofiber penetration depths exhibited a three-step weight loss pattern similar to that of pure PVA. Although PD-0 with full penetration of PCL nanofibers showed higher thermal stability at the second stage (200–340 °C), the improvement in the thermal stability for all PVA/PCL composite films was not prominent and the crystalline PCL nanofibers did not effectively improve the thermal properties of the PVA film. This can be related to the poor chemical interaction of PVA and the higher thermal stability of the PCL nanofibers. The incorporation of crystalline PCL nanofibers with higher thermal conductivity owing to the fast thermal transfer can degrade the thermal stability of the composite films [63].

### 3.6. Oxygen Barrier Properties

Table 2 shows the OTR of the pure PVA and PVA/PCL composite films. The pure PVA film had an OTR of 5.5 cc/m^2^·day, similar to previous results [64,65]. Regardless of the predrying time and the penetration depth, the introduction of PCL nanofibers into the PVA film significantly decreased (*p* < 0.05) the OTR from 1.6 to 3.6 cc/m^2^·day. PD-10 exhibited the lowest OTR of 1.6 cc/m^2^·day and PD-5–PD-20 exhibited decreasing OTR of 1.6–1.9 cc/m^2^·day. 

The gas barrier properties of the polymer films are influenced by the chemical structure and morphology of the matrix polymer and composite films with organic or inorganic fillers [66,67]. Electrospun PCL nanofibers have crystalline structures that impede the diffusion of oxygen molecules and provide a tortuous and obstructed path for oxygen permeation, promoting oxygen barrier properties [68]. Moreover, the hydrophobicity of the PCL nanofibers can result in their poor interactions and incompatibility with hydrophilic PVA, creating voids or micropores and deteriorating the oxygen barrier properties [68,69]. These two opposing aspects should be considered in the oxygen barrier properties of PVA/PCL composite films. The minimal improvement in the oxygen barrier properties of PD-0 can be attributed to the pores or voids at the interface of the PCL nanofibers and PVA matrix. Additionally, the simple lamination of PCL on the PVA film and the chemical incompatibility of crystalline PCL nanofibers with the PVA matrix decrease the oxygen barrier properties of PD-30.

## 4. Conclusions

In this study, PVA/PCL composite films with enhanced mechanical strength, excellent barrier properties, WCAs, and physical binding between hydrophobic nanofibers and hydrophilic polymer matrices were fabricated using a novel surface modification approach. The control of the predrying conditions and optimization of the electrospinning process were key steps to realizing the performance of the resulting films. This work focused on the effect of the predrying time on penetrating PCL nanofibers, revealing the relationship between the penetration depth and predrying time. Further, this finding was related to the varying residual solvent contents of the predried PVA film. A higher solvent content in the PVA film increased the polymer chain mobility, facilitating the PCL nanofibers to penetrate the PVA film. The difference in the penetration depth exerted a notable influence on the physical properties. The results revealed a notable correlation between the predrying time and WCA of the PCL/PVA composite films owing to the higher exposure of the PCL nanofibers on the film surface. The PVA/PCL composite films, particularly PD-15, exhibited substantial improvements in the mechanical properties owing to the presence of PCL nanofibers and reduced pore formation. Regardless of the predrying time, the incorporation of PCL nanofibers enhanced the oxygen barrier properties. Therefore, the optimization of the predrying conditions and precise adjustment of electrospun nanofibers can improve the physical binding and modify the surface chemistry of film substrates for hydrophilic polymers, expanding their packaging applications.

## Figures and Tables

**Table 1:** Solvent contents of the samples with varying predrying times.

Sample	Predrying Time (min)	Electrospinning Time (s)	Residual Solvent Content Measured by TGA (%)
PD-0	0	30	79.4 ± 5.6 ^a^
PD-5	5	30	73.6 ± 5.8 ^ab^
PD-10	10	30	66.5 ± 4.0 ^bc^
PD-15	15	30	63.5 ± 3.5 ^c^
PD-20	20	30	15.0 ± 0.2 ^d^
PD-30	30	30	5.8 ± 0.3 ^e^

Data reported are mean ± standard deviations. Any means in the same column followed by different superscripts are significantly (*p* < 0.05) different by Duncan’s multiple range test.

**Table 2:** Mechanical properties and OTR of the pure PVA and PVA/PCL composite films.

Sample Code	Tensile Strength (MPa)	Elongation at Break (%)	OTR (cc/m^2^·day)
Pure PVA	7.4 ± 0.5 ^e^	290.0 ± 3.1 ^c^	5.5 ± 0.1 ^a^
PD-0	33.5 ± 2.5 ^d^	139.8 ± 12.2 ^e^	3.6 ± 0.3 ^b^
PD-5	62.5 ± 3.8 ^b^	277.4 ± 21.1 ^c^	1.8 ± 0.6 ^c^
PD-10	62.9 ± 4.2 ^b^	295.4 ± 11.4 ^bc^	1.6 ± 0.6 ^c^
PD-15	77.4 ± 7.9 ^a^	340.5 ± 22.9 ^a^	1.9 ± 0.5 ^c^
PD-20	52.5 ± 2.7 ^c^	330.8 ± 35.5 ^ab^	1.9 ± 0.3 ^c^
PD-30	47.8 ± 5.5 ^c^	230.0 ± 20.4 ^d^	3.4 ± 0.9 ^b^

Data reported are mean ± standard deviations. Any means in the same column followed by different superscripts are significantly (*p* < 0.05) different by Duncan’s multiple range test.

## Data Availability

The original contributions presented in the study are included in the article, further inquiries can be directed to the corresponding author.
